# Clinical Evaluation of Nuclear Imaging Agents in Breast Cancer

**DOI:** 10.3390/cancers14092103

**Published:** 2022-04-23

**Authors:** Ziqi Li, Mariam S. Aboian, Xiaohua Zhu, Bernadette Marquez-Nostra

**Affiliations:** 1 Department of Nuclear Medicine, Tongji Hospital, Tongji Medical College, Huazhong University of Science and Technology, Wuhan 430030, China; lzqhhc@tjh.tjmu.edu.cn; 2 PET Center, Department of Radiology and Biomedical Imaging, Yale University, P.O. Box 208048, New Haven, CT 06520, USA; mariam.aboian@yale.edu

**Keywords:** breast cancer, clinical, precision imaging, first-in-human, radiopharmaceuticals

## Abstract

**Simple Summary:**

Breast cancer is currently the most common type of diagnosed cancer worldwide. Noninvasive imaging of therapeutic targets or biomarkers for breast cancer has the potential to contribute to precision medicine, where targeted therapy is needed. Positron emission tomography (PET) or single-photon emission tomography (SPECT) imaging with radiolabeled probes has the potential to play an important role in the molecular profiling of therapeutic targets in vivo for the selection of patients who are likely to respond to corresponding targeted therapy. This review covers recent clinical investigations with noninvasive imaging agents in breast cancer. We reviewed 17 clinical studies on PET or SPECT agents that target 10 receptors in breast cancer.

**Abstract:**

Precision medicine is the customization of therapy for specific groups of patients using genetic or molecular profiling. Noninvasive imaging is one strategy for molecular profiling and is the focus of this review. The combination of imaging and therapy for precision medicine gave rise to the field of theranostics. In breast cancer, the detection and quantification of therapeutic targets can help assess their heterogeneity, especially in metastatic disease, and may help guide clinical decisions for targeted treatments. Positron emission tomography (PET) or single-photon emission tomography (SPECT) imaging has the potential to play an important role in the molecular profiling of therapeutic targets in vivo for the selection of patients who are likely to respond to corresponding targeted therapy. In this review, we discuss the state-of-the-art nuclear imaging agents in clinical research for breast cancer. We reviewed 17 clinical studies on PET or SPECT agents that target 10 different receptors in breast cancer. We also discuss the limitations of the study designs and of the imaging agents in these studies. Finally, we offer our perspective on which imaging agents have the highest potential to be used in clinical practice in the future.

## 1. Introduction

Breast cancer is currently the most common type of diagnosed cancers worldwide, accounting for about 30% of all new diagnoses in female cancers each year [1]. Breast cancer is a heterogeneous disease, where the tumorigenicity, metastatic potential, and sensitivity to treatments differ greatly among patients [2,3]. Furthermore, the status of predictive biomarkers for treatment may also evolve during tumor progression. For example, the discordant expression between primary and metastatic lesions in breast cancer of human epidermal growth factor 2 (HER2), estrogen receptor (ER), and progesterone receptor (PR) has been extensively reported [4]. Thus, heterogeneity of receptor expression seriously impedes the successful clinical management of breast cancer.

According to the latest National Comprehensive Cancer Network (NCCN) guidelines, most breast cancer patients will undergo mammography, computed tomography (CT), magnetic resonance imaging (MRI), or a bone scan before treatment. When anatomic imaging results are unclear, some patients would receive [^18^F]-fluorodeoxyglucose ([^18^F]FDG)-positron emission tomography (PET) scans to identify metabolically active tumor lesions [5]. [^18^F]FDG-PET is the standard of care for staging locally advanced and inflammatory breast cancer. It is also used for the restaging of recurrence [6]. Because of the limitations in detection of early axillary node involvement and micrometastases, [^18^F]FDG-PET was not suggested for use in the staging of patients with early breast cancer [7]. More importantly, these imaging techniques cannot assess the heterogeneity of therapeutic target expression within a patient. 

Currently, tissue analysis using immunohistochemistry (IHC) and fluorescent in situ hybridization (FISH) are still the most-used methods to detect HER2, ER, and PR in treatment planning. However, the procurement of tissue samples is limited by the need for invasive biopsy. Tissue analysis is then limited by the heterogeneity of antigen expression and differences in the interpretation of results by different pathologists [8]. To circumvent these limitations, researchers have focused on the development of novel noninvasive imaging agents to detect and quantify therapeutic target expression in vivo. Unlike a biopsy, nuclear imaging using PET or single-photon emission tomography (SPECT) allows for noninvasive, quantitative, and whole-body assessment of receptor status. Noninvasive imaging by PET or SPECT is currently playing a role in individualizing the patient’s treatment regimen. For instance, the first U.S. Food and Drug Administration (FDA)-approved receptor-targeted PET radiotracer for breast cancer patients is [^18^F]-fluoroestradiol ([^18^F]FES), which is being used for treatment planning with ER-targeted agents [9]. Additionally, SPECT/CT offers enhanced preoperative visualization of sentinel lymph nodes, with further implementation into personalized surgical approach [10]. The rise in the availability of new targeted treatments (e.g., antibody drug conjugates, targeted radiotherapy, and immunotherapy) warrants the development of corresponding imaging agents to predict or monitor response to treatment.

Theranostics, which combines therapeutic and diagnostic agents, is gaining momentum in the era of precision medicine for other types of cancer. One example is the striking development of [^68^Ga]Ga-PSMA, which led to the boom of PSMA-targeted radioligands, including FDA-approved [^177^Lu]Lu-PSMA-617 (Pluctivo), for the treatment of metastatic castration-resistant prostate cancer [11,12]. Nowadays, several FDA-approved theranostic pairs, such as [^68^Ga]DOTATATE and [^177^Lu]DOTATATE for neuroendocrine tumors, are used in clinical nuclear medicine practice [13,14]. However, FDA-approved theranostic pairs for breast cancer are currently limited. Thus, the invention of nuclear imaging agents that could be used in theranostics for breast cancer is also warranted to help with patient selection, treatment planning, and monitoring response to treatment.

Some nuclear imaging agents have limitations, such as high uptake in the liver or kidney. In breast cancer, the liver, bone, lung, and brain are typical sites for metastases. Thus, it is important that the tracer of interest has the sensitivity to detect its target in these organs. For these reasons, clinical studies are key steps in identifying tracer-specific limitations to help guide the development of better tracers. This review covers state-of-the-art and emerging strategies for nuclear imaging with novel probes for breast cancer in clinical research in the past seven years. Table 1 summarizes the targets for and properties of all tracers in this review. The subsections of this review are organized by the different targets for the tracers.

## 2. Tracers for Specific Targets

### 2.1. Human Epidermal Growth Factor 2 (HER2)

HER2 is one of the most extensively studied receptors for breast cancer. HER2 is overexpressed in almost 25% of breast cancers, and is associated with increased recurrence, distant metastasis, and shorter survival [32]. Several probes against HER2 have been labeled for nuclear imaging and/or therapy. HER2-targeted probes in nuclear medicine prior to 2018 were previously reviewed [33]. Here, we summarize new findings in clinical studies of HER2 imaging from 2018 to 2022.

Trastuzumab (Herceptin; Genentech, South San Francisco, CA, USA) was the first FDA-approved, humanized monoclonal antibody against HER2. Trastuzumab is widely used for the treatment for HER2-positive breast cancer. In identifying patients who may benefit from this antibody, trastuzumab was conjugated to the CHX-A″-DTPA chelator and radiolabeled with ^111^In to obtain [^111^In]In-CHX-A″-DTPA-trastuzumab [15]. The safety and biodistribution of [^111^In]In-CHX-A″-DTPA-trastuzumab were evaluated in 11 patients, of which 8 patients had metastatic breast cancer. After administering the mean dose of 175 MBq of [^111^In]In-CHX-A″-DTPA-trastuzumab, patients underwent a single (n = 5) or multiple ɣ-camera (n = 6) and/or SPECT (n = 8) imaging at different timepoints between 2–168 h. Tumor-to-background ratios (T/B) of greater than 1.5 were achieved at all timepoints. In the 8 patients with metastatic breast cancer, the results of visual and semiquantitative analyses were concordant with tissue profiling. Typically, preinjection of unlabeled antibody is needed to saturate binding to Fc receptors in normal organs and to allow the radiolabeled antibody to reach its target receptor in the tumor [33]. However, [^111^In]In-CHX-A″-DTPA-trastuzumab demonstrated excellent imaging characteristics without preinjection of unlabeled trastuzumab. The safety and sensitivity of [^111^In]In-CHX-A″-DTPA-trastuzumab suggested that it can potentially be used in the clinic as a diagnostic tool for HER2-positive tumors in breast cancer. 

Since HER2 expression can change over the course of the disease, a noninvasive method to assess HER2 status in vivo would be beneficial for patients being treated with HER2-targeted therapy. For example, the loss of the HER2 extracellular domain would result in no antibody binding and would make these treatments ineffective [34]. Several studies showed that PET imaging with [^64^Cu]Cu-DOTA-trastuzumab could be used to visualize HER2 in primary breast cancers, lymph node metastases, and lung metastases [35]. The concordance of [^64^Cu]Cu-DOTA-trastuzumab-PET with HER2-IHC analysis was evaluated in 38 patients with breast cancer [17]. PET/CT scans were carried out at 48 h after injection of approximately 130 MBq of [^64^Cu]Cu-DOTA-trastuzumab. The SUV_max_ was found to be 2.6 ± 0.9 for HER2-positive, and 1.4 ± 0.9 for HER2-negative breast tumors. The result showed that the SUV_max_ values in each lesion were correlated with their HER2-IHC scores (R = 0.619). [^64^Cu]Cu-DOTA-trastuzumab PET imaging identified 15 out of 18 HER2-positive (based on IHC) tumors and 15 out of 17 HER2-negative tumors. The sensitivity, specificity, and accuracy were 83.3%, 88.2%, and 85.7%, respectively. This study demonstrated that [^64^Cu]Cu-DOTA-trastuzumab-PET could supplement IHC analysis in the identification of HER2-positive primary and metastatic tumors. 

Admittedly, antibodies often demonstrate high affinity and selectivity for their antigens, which made antibodies desirable as imaging probes. However, they have some limitations, such as their relatively poor heat stability, poor tumor penetration relative to smaller scaffolds, high uptake in the liver, and slow pharmacokinetic properties for imaging purposes [36]. As a viable and sometimes superior alternative to antibodies, affibodies are a class of engineered protein scaffolds that overcome some limitations of antibodies [37]. The small size of affibody molecules (∼7 kDa) is a favorable property for diagnostic imaging, due to their relatively fast clearance from the bloodstream, high affinity for their target proteins, and relatively lower uptake in the liver compared with antibody probes. Z_HER2:342_ is an HER2-binding affibody that does not interact with therapeutic anti-HER2 antibodies. Both preclinical and clinical studies were conducted with the probe, [^68^Ga]Ga-NOTA-MAL-Cys-MZ_HER2:342_ [16]. PET imaging was performed in two patients with breast cancer at 60 min postinjection of 74 MBq [^68^Ga]Ga-NOTA-MAL-Cys-MZ_HER2:342_. The tumor uptake of the tracer was significantly higher in the HER2-positive breast cancer patients than in those with HER2-negative disease with SUV_max_ of 2.16 ± 0.27 and 0.32 ± 0.05, respectively. This novel probe might be valuable in quantifying HER2 expression in vivo at clinically desirable imaging timepoints. However, the SUV_max_ in the kidney in the two patients were 12.15 ± 2.74 and 10.27 ± 2.29, respectively. This high renal accumulation was similar to that of other radiometal-labeled affibody molecules [38,39,40]. Renal toxicity has yet to be evaluated. Despite the high background signal in the kidney, renal metastases are rare in breast cancer; therefore, high uptake of the tracer in the kidney may not be an issue for diagnostic purposes with this PET tracer. 

**Figure 1:**
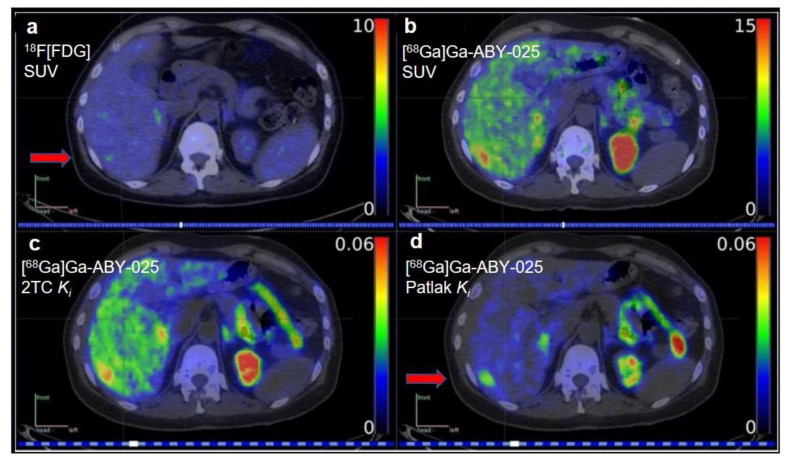
Patlak *K_i_* images of [^68^Ga]Ga-ABY-025 provided good visualization of liver metastases and mitigated nonspecific background uptake in this organ relative to standardized uptake value (SUV) images. (**a**) [^18^F]FDG-PET, (**b**) [^68^Ga]Ga-ABY-025-PET, (**c**) parametric images of 2-tissue compartment (2TC) *K_i_*, and (**d**) Patlak *K_i_* in a breast cancer patient with multiple small liver metastases in the same patient. (Reprinted from Ref. [18]).

The second generation Affibody^®^ molecule, ABY-025, has been improved by further modification of the nonbinding surface of Z_HER2:342_, and it can bind selectively to HER2 receptors with higher thermal stability and hydrophilicity than Z_HER2:342_ [36]. ^68^Ga-labeled affibody ABY-025 has also been investigated as an HER2-targeted imaging agent [41]. The Phase I clinical study of [^68^Ga]Ga-ABY-025-PET/CT in the detection and quantification of HER2 expression demonstrated high stability, fast blood clearance, and high reproducibility of the radiotracer [42]. In this study, kinetic modeling was used to quantify tracer uptake. Tracer kinetic models are the mathematical models that describe the time-varying distribution of radiotracers in the body [43]. Compared with SUV, kinetic modeling may provide a more accurate quantification of the tracer uptake in organs of interest [44]. Kinetic modeling may impact precision medicine through better estimations for the dosing of therapeutic agents, a more accurate dosimetry for radioligand therapies, and more accurate estimations of adverse events in nontarget organs. Alhuseinalkhudhur et al. explored kinetic modeling to analyze the relationship between the rates of [^68^Ga]Ga-ABY-025 uptake and HER2 expression in the tumor [18]. Sixteen patients with metastatic breast cancer underwent dynamic [^68^Ga]Ga-ABY-025-PET/CT imaging from 0–45 min postinjection. To test the reproducibility of [^68^Ga]Ga-ABY-025, 5 of the 16 patients underwent two PET scans with [^68^Ga]Ga-ABY-025. An [^18^F]FDG-PET examination was performed within 14 days prior to the first [^68^Ga]Ga-ABY-025-PET for all these patients. Parametric images of tracer delivery (*K_1_*), irreversible binding (*K_i_*), and SUVs were calculated. Two-tissue-compartment (2TC) model and Patlak analyses were both used to create parametric images. The results showed that the *K_i_* values agreed very well with the volume-of-interest (VOI)-based gold standard (R^2^ > 0.99, *p* < 0.001). SUVs in metastases at 2 h and 4 h post-injection were highly correlated with *K_i_* values derived from both the 2TC model and Patlak methods (R^2^ = 0.87 and 0.95, both *p* < 0.001). High retest reliability was shown by the parametric image-based *K_i_* values (Pearson’s r ≥ 0.92, n = 5). Parametric imaging provided good visualization and mitigated nonspecific background uptake in the liver (Figure 1). This study provided the proof-of-concept testing of tracer kinetic modeling in clinical imaging. Kinetic modeling could be very useful in quantifying tracer uptake in small metastatic lesions in organs where high background activity could be present. 

In addition to affibodies, designed ankyrin repeat proteins (DARPins), another kind of engineered protein scaffold, are promising probes for HER2 imaging [45]. DARPins hold the ideal characteristics of imaging agents, including relatively small molecular weight (14–18 kDa), high binding affinity, high specificity to their respective targets, high chemical and thermal stability, and potentially low production costs [46]. Bragina et al. conducted a first-in-human study to evaluate the safety and distribution of [^99m^Tc]Tc-(HE)3-G3 in patients with primary breast cancer [19]. Twenty-eight patients were enrolled in the trial. Three cohorts of patients with primary breast cancer were injected with 1-, 2-, or 3- mg protein doses of [^99m^Tc]Tc-(HE)3-G3 (287 ± 170 MBq). Each cohort included at least four patients with HER2-negative and five patients with HER2-positive tumors. SPECT scans were performed at 2-, 4-, 6-, and 24- h after injection. No side effects were observed during imaging and up to 7 days after injection. Clear visualization of tumors could be observed as early as 2 h after injection. At 2 h and 4 h after injection, the tumor–to–contralateral site ratios for HER2-positive tumors were significantly higher than those for HER2-negative tumors (*p* < 0.05). The hepatic uptake decreased after increasing the injected mass dose from 1 to 3 mg. Thus, an injected protein mass dose between 2–3 mg is optimal for [^99m^Tc]Tc-(HE)3-G3. Taken together, the desirable properties of this agent support its further development for other imaging modalities, or even as a therapeutic agent.

^111^In-, ^64^Cu-, and ^68^Ga-labeled probes for HER2 have demonstrated good sensitivity and specificity for the detection of HER2-positive metastatic breast cancer. These probes include antibodies used for therapy, such as trastuzumab, and smaller probes such as affibodies. Further studies, such as studies of the direct correlations between imaging and pathology, as well as evaluations of these tracers in patients with brain metastases, are needed prior to implementing these tracers in clinical practice. If successful, these tracers could select patients who will likely respond to trastuzumab or other HER2-targeted treatments, and to monitor HER2 expression levels in multi-focal metastatic disease. Thus, the potential for HER2-targeted imaging agents could impact the treatment of all HER2-positive metastatic lesions, as opposed to the assumption that all metastatic lesions are HER2-positive based on analysis of biopsied tissue.

### 2.2. Hormone Receptors

Hormone receptors play a key role in regulating the growth and differentiation of breast epithelium, and they are prognostic indicators for positive treatment outcomes in breast cancer. ER is expressed in 80% of breast cancer cases. Of those patients who are ER-positive, 65% are also PR-positive [47]. Both receptors are strong predictive markers of response to endocrine therapy. It could be beneficial to assess the status of ER and PR to guide decisions on adjuvant therapy and to evaluate medical prognosis for breast cancer. In addition, monitoring endocrine treatment response with tracers that target ER or PR would be useful in determining whether endocrine treatment needs to continue or whether alternative treatment strategies would be needed. Hence, a reproducible noninvasive diagnostic technique to map hormone receptor expression would be clinically valuable.

#### 2.2.1. Estrogen Receptor (ER)

The quantification of ER may be helpful in dictating the appropriateness of hormonal therapy. The use of [^18^F]FES-PET to map ER has some drawbacks, including rapid metabolism, which makes quantitative analysis complicated, and its high background uptake in the liver [48]. Thus, new tracers that provide more stable imaging of ER receptors than [^18^F]FES are needed. 

4-Fluoro-11β-methoxy-16α-[^18^F]fluoroestradiol ([^18^F]4FMFES) is one of the series of 11β-methoxy- or A-ring fluorine-substituted [^18^F]FES derivatives used to overcome the shortcomings of [^18^F]FES. Paquette et al. conducted a Phase II clinical trial to compare the imaging quality of [^18^F]4FMFES with that of [^18^F]FES in ER-positive breast cancer patients [21]. [^18^F]4FMFES and [^18^F]FES-PET/CT scans were done sequentially (within one week) and in random order in 31 patients with ER-positive breast cancer. In addition to the 96 ER-positive lesions identified by [^18^F]FES, [^18^F]4FMFES succeeded in detecting 9 additional lesions, which were confirmed as true-positives via biopsy or [^18^F]FDG-PET/CT. The two tracers exhibited a similar tumor SUV_max_; however, [^18^F]4FMFES showed less overall background than [^18^F]FES, especially in the mediastinal region. Hence, [^18^F]4FMFES-PET showed higher detection rates and better sensitivity than [^18^F]FES in this study. One explanation could be that the structure of [^18^F]4FMFES has lower nonspecific binding to plasma globulins, which improves its in vivo stability. 

Tamoxifen is the oldest ER modulator approved by the FDA for the treatment of patients diagnosed with ER-positive breast cancer [49]. [^99m^Tc]Tc-tamoxifen for SPECT imaging is reported as a potential probe that is more cost-effective than [^18^F]FES-PET imaging for mapping ER in vivo [50]. In a case report, a 62-year-old woman, who had lumpectomy 4 years before the study, underwent [^99m^Tc]Tc-tamoxifen imaging [20]. This patient was diagnosed with ER-expressing invasive ductal carcinoma. After injection of 311 MBq of [^99m^Tc]Tc-tamoxifen, serial images were acquired from 0 to 19 h. [^99m^Tc]Tc-tamoxifen was taken up in the chest wall, right axial lymph nodes, lung nodule, and supraclavicular lymph nodes, which also showed positivity by [^18^F]FDG-PET. Compared with [^18^F]FES, the synthesis of [^99m^Tc]Tc-tamoxifen is more cost-effective as it does not require an on-site medical cyclotron facility. In addition, tamoxifen is an FDA-approved drug for adjuvant therapy in ER-expressing breast cancer patients. Therefore, this study laid the foundation for the significance of imaging agents with radiolabeled tamoxifen as potential biomarkers of patients’ responses to tamoxifen. 

Overall, ER-targeted imaging tracers include the FDA-approved [^18^F]FES, [^99m^Tc]Tc-tamoxifen, and [^18^F]4FMFES. These tracers allow visualization of ER within primary and metastatic tumors and can provide information on whether a patient will respond to ER-targeted therapy. 

#### 2.2.2. Progesterone Receptor (PR)

PR is an estrogen-regulated protein and can be an indicator of ER functionality. Patients with PR-positive breast cancer are treated with estrogen receptor inhibitors. It is reported that in ER-positive breast cancer patients, endocrine therapy response rates were higher in PR-positive patients than in PR-negative patients, as the co-expression of ER and PR is indicative of a functionally intact estrogen response pathway [51,52]. Thus, PR-targeted PET imaging has the potential to predict responses to endocrine therapy. The most studied PR-targeted radiopharmaceutical is [^18^F]-fluorofuranyl norprogesterone ([^18^F]FFNP). Dehdashti et al. recently investigated whether the change in [^18^F]FFNP uptake in a tumor after estradiol challenge relative to baseline could predict responses to endocrine therapy in women with ER-positive breast cancer [22]. Forty-three women with locally recurrent or metastatic breast cancer were enrolled in this study. All tumors were ER-positive. The patients underwent two [^18^F]FFNP scans, one before and one immediately following the one-day estradiol challenge. Following PET studies, the patients underwent various types of endocrine therapy. Twenty-eight patients (65%) responded to treatment and had no disease progression within 6 months. All of them showed a post-challenge increase in [^18^F]FFNP uptake in the tumor. In contrast, the remaining 15 patients who progressed within 6 months had no increase in tracer uptake in the tumor. Thus, the tracer demonstrated 100% sensitivity and specificity (*p* < 0.0001). Notably, [^18^F]FFNP uptake in the tumor at baseline did not differ significantly between responders and nonresponders. This study demonstrated that the change in [^18^F]-FFNP uptake in a tumor after estradiol challenge is highly predictive of responses to endocrine therapy in women with ER-positive breast cancer.

#### 2.2.3. Androgen Receptor (AR)

About 70–80% of all breast cancer is AR-positive, and AR has emerged as a possible target for breast cancer therapy [53]. 16β-[^18^F]fluoro-5α-dihydrotestosterone ([^18^F]FDHT) PET/CT was developed to assess the AR status in tumor lesions and showed a good correlation between tracer uptake and AR expression in biopsied tissues in several studies [54]. To examine the interobserver variability of [^18^F]FES and [^18^F]FDHT-PET in breast cancer, 10 patients with ER-positive metastatic breast cancer were included in a prospective, two-center clinical study [23]. Doses of 200 MBq (±10%) of [^18^F]FES and [^18^F]FDHT were injected on separate days within 2 weeks. A PET/CT scan was performed after 60 min. In addition, high-resolution, contrast-enhanced CT scans of chest-abdomen and bone were performed within 6 weeks for comparison, resulting in the identification of 121 total lesions. [^18^F]FES-PET showed high positive and negative interobserver agreement of 84% and 83%, respectively, by visual analysis. On the contrary, low T/B ratios were found for [^18^F]FDHT-PET, with 49% positive and 74% negative interobserver agreement. [^18^F]FES-PET showed an excellent intraclass correlation coefficient (ICC) for SUV_max_ (ICC = 0.98) and SUV_peak_ (ICC = 0.97), and a good ICC for SUV_mean_ (ICC = 0.89), while the ICC of SUV_max_, SUV_peak_ and SUV_mean_ were 0.78, 0.76, and 0.75 for [^18^F]FDHT, respectively. As a result of the low AR expression in breast cancer patients, [^18^F]FDHT-PET showed relatively lower visual agreement than [^18^F]FES in this study. Further studies are required in view of the good quantitative agreement between observers. Overall, AR-targeted imaging with [^18^F]FDHT does not show high positive interobserver agreement, and its translation into clinical practice may be limited.

### 2.3. Other Receptors

#### 2.3.1. Integrin Alpha v Beta 3 (αvβ3)

Except for the most studied receptors, HER2, ER, and PR, there are still many markers that are highly expressed in breast cancer, which have been adopted as imaging targets. Integrin αvβ3 is a member of the integrin superfamily of adhesion molecules, which plays a critical role in tumor angiogenesis and metastasis. Currently, there are no FDA-approved therapeutic agents targeting integrin αvβ3, so this imaging agent would be limited to the application of tumor detection at this time. 

Over the past decade, Arg-Gly-Asp (RGD) derivatives, which specifically target integrin αvβ3, have been radiolabeled and investigated for noninvasive imaging of tumors in both preclinical and clinical studies [55,56]. Imaging integrin αvβ3 could help with predicting disease prognosis. A peptide based on three polyethylene glycol spacersarginine-glycine-aspartic acid (3PRGD2) is a new cyclic RGD variant. To compare the diagnostic value of [^99m^Tc]Tc-3PRGD2 imaging with [^18^F]FDG-PET/CT in the diagnosis and staging of breast cancer, 42 women with suspected breast cancer were enrolled in the trial [24]. For visual analysis of breast lesions, the sensitivity of PET imaging with [^99m^Tc]Tc-3PRGD2 is higher than that with [^18^F]FDG (97.4% vs. 87.5%), while the specificity and accuracy of [^99m^Tc]Tc-3PRGD2 are lower than those of [^18^F]FDG (*p* > 0.05). For axillary lymph node metastasis, the sensitivity of [^99m^Tc]Tc-3PRGD2 is lower than that of [^18^F]FDG (78.05% vs. 99.36%, *p* > 0.05). Although [^99m^Tc]Tc-3PRGD2 imaging was less sensitive than [^18^F]FDG-PET in detecting lymph node metastatic lesions, it appears to be valuable for the diagnosis and staging of breast cancer because of its high sensitivity for visual analysis of primary breast lesions. It also should be noted that the state of integrin αvβ3 differs with pathological subtype and clinical stage [57]. Thus, the use of this tracer needs to be defined in more studies.

#### 2.3.2. Gastrin-Releasing Peptide Receptor (GRPR)

The gastrin-releasing peptide receptor has been found to be overexpressed in many types of tumor cells, including breast cancer cells [58]. In vitro studies suggested a positive correlation between ER and GRPR expression [59]. This study demonstrated that it is possible to monitor ER status by imaging GRPR expression in patients.

GRPR-PET/CT imaging may provide information about the ER status of breast cancer. RM2, a GRPR antagonist, was recently shown to be safe for use in humans [60]. ^68^Ga-labeled [^68^Ga]Ga-RM2 showed a good diagnostic accuracy for a primary prostate carcinoma [61]. Stoykow et al. designed a clinical study to verify its value in breast cancer patients [25]. Compared with normal breast tissue, 13 out of 18 tumors were clearly visualized by increased [^68^Ga]Ga-RM2 uptake in 15 female patients with primary breast cancer. IHC confirmed that all [^68^Ga]Ga-RM2-PET-positive lesions were ER and PR positive. They determined that [^68^Ga]Ga-RM2 uptake correlated well with ER expression (Spearman’s ρ = 0.70, *p* = 0.0013), which suggested that the radiotracer had high sensitivity for ER-positive tumors. The limitations of this study include a lack of comparison with [^18^F]FDG-PET, and only patients with known breast cancer were included in the study. 

RM26 (D-Phe-Gln-Trp-Ala-Val-Gly-His-Sta-Leu-NH2) is another GRPR antagonist with high affinity. The tracer [^68^Ga]Ga-NOTA-RM26 was shown to be safe and useful in prostate cancer patients [62]. Zhang et al. conducted a small prospective study to evaluate [^68^Ga]Ga-NOTA-RM26 in patients with suspicious breast lesions [26]. Thirty-five women suspected of breast cancer by mammography or ultrasonography were injected with [^68^Ga]Ga-NOTA-RM26 within 1 week before surgery. Thirty-four patients were diagnosed with breast cancer by biopsy. [^68^Ga]Ga-NOTA-RM26 had a positive correlation with ER expression (*p* = 0.006). The SUV_max_ in the ER-positive breast cancer (SUV_max_ = 4.97 ± 1.89) was significantly higher than that in patients with ER-negative breast cancer (SUV_max_ = 2.78 ± 0.65, *p* < 0.001). [^68^Ga]Ga-NOTA-RM26 accumulated in normal breast tissue, and the SUV_max_ was found to be variable during the menstrual cycle; it is higher in the secretory phase (SUV_max_ = 2.27 ± 0.71) than in the nonsecretory phase (SUV_max_ = 1.59 ± 0.49, *p* = 0.017) or at postmenopause (SUV_max_ = 1.43 ± 0.44, *p* = 0.002). When excluding the cases in the secretory phase, the sensitivity, specificity, and accuracy of this probe was 100.0%, 90.9%, and 95.5%, respectively. This result suggested that the best time for [^68^Ga]Ga-NOTA-RM26-PET to monitor ER expression in breast cancer was during the proliferative phase in premenopausal women. This clinical trial is an exploration of the correlation between the SUV of [^68^Ga]Ga-NOTA-RM26 in breast tissue and the menstrual cycle. Further studies in the same patients during the menstrual cycle will be needed to obtain more definitive conclusions.

#### 2.3.3. Chemokine Receptor Type 4 (CXCR4)

**Figure 2:**
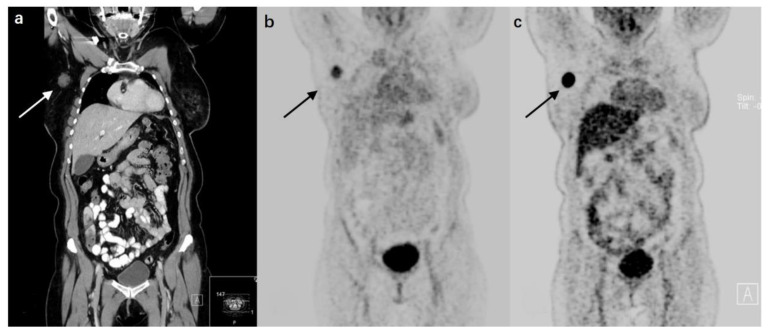
A 67-year-old patient with a nodal recurrence at 22 months after treatment from primary breast cancer. (**a**) Coronal CT reconstruction shows a contrast-enhancing lymph node metastasis with a diameter of 2.1 cm in the right axillary region. (**b**) The lesion is visually detectable on [^68^Ga]Pentixafor-PET (SUV_max_ = 4.0). (**c**) The lesion has a significantly higher [^18^F]FDG uptake (SUV_max_ = 24.4). (Reprinted from Ref. [27]).

The chemokine receptor CXCR4 is expressed in many breast cancers, and has an important role in the migration, invasiveness, metastasis, and proliferation of tumors [63]. Several CXCR4 inhibitors or antagonists, such as AMD3100, Pentixafor, and T140, have been radiolabeled and used to image CXCR4 in small animals [64,65]. To evaluate the use of [^68^Ga]Pentixafor in detecting breast cancer, 18 patients underwent [^68^Ga]Pentixafor PET/CT or PET/MR, including 13 patients with a first diagnosis of breast cancer, four patients with recurrent disease after primary breast cancer, and one patient with axillary lymph node metastasis of unknown primary [27]. Nine of the 13 primary tumors were visually detected with [^68^Ga]Pentixafor, and all 5 metastases could be visually identified. Eight of them (4 recurrent breast cancer patients and 4 primary breast cancer patients) additionally received an [^18^F]FDG-PET within 2 weeks after administration of [^68^Ga]Pentixafor. Higher SUV_max_ of [^18^F]FDG were observed in all cases, compared with [^68^Ga]Pentixafor (mean SUV_max_ of 16.2 vs. mean SUV_max_ of 3.6; *p* < 0.05).(Figure 2) This study did not reveal any significant correlation between [^68^Ga]Pentixafor uptake and breast cancer prognostic factors (ER, PR, or HER2 status), proliferation index, or tumor grade. Moreover, [^68^Ga]Pentixafor uptake seemed to vary with histological tumor types. Since CXCR4 signaling mechanistically drives ER-positive breast cancers to metastatic and endocrine therapy-resistant phenotypes, PET imaging with [^68^Ga]Pentixafor might play a role in providing spatiotemporal information over the course of endocrine therapy [66].

#### 2.3.4. Prostate-Specific Membrane Antigen (PSMA)

PSMA is one of the most studied targets for imaging and therapy of prostate cancer. PSMA has been reported to be overexpressed in the neovasculature of not only malignant tumors, including prostate cancer and breast cancer, but also in benign tumors or in inflammatory lesions. Moreover, PSMA is an important biomarker of angiogenesis [67]. [^68^Ga]Ga -PSMA-11 (HBED-CC) is the first FDA-approved PSMA-targeted PET imaging agent for men with prostate cancer [68]. To discover its potential value in breast cancer, [^68^Ga]Ga-PSMA-HBED-CC-PET/CT was also evaluated in 19 breast cancer patients [28]. Eighty-one lesions were identified in this group, of which 84% were detected by [^68^Ga]Ga-PSMA-HBED-CC-PET/CT. Seven patients underwent both [^68^Ga]Ga-PSMA-HBED-CC- and [^18^F]FDG-PET/CT, with [^18^F]FDG-PET detecting 35 lesions and [^68^Ga]Ga-PSMA-HBED-CC-PET detecting 30 lesions. Six of the [^18^F]FDG-positive lesions were negative on [^68^Ga]Ga-PSMA-HBED-CC-PET, while one of the [^68^Ga]Ga-PSMA-HBED-CC-positive lesions was negative on [^18^F]FDG-PET. Moreover, this study confirmed that the PSMA expression on breast cancer concurred with IHC analysis [69]. 

It is interesting to note a weak correlation and statistically significant P value between the SUVs of these two tracers in the tumor (r = 0.407, *p* = 0.015). Sathekge et al. suggested that there is a relationship between tumor metabolism as assessed by [^18^F]FDG uptake and tumor angiogenesis as assessed by [^68^Ga]Ga-PSMA-HBED-CC uptake. [^18^F]FDG uptake has been previously shown to correlate with pathologic angiogenesis biomarkers, but not all studies found the same correlation [70]. We believe that this study was not sufficiently powered (seven patients) for a rigorous comparison [71]. If future studies confirm the relationship between PSMA expression and tumor angiogenesis, PET imaging with [^68^Ga]Ga-PSMA-HBED-CC could be explored for predicting and monitoring responses to antiangiogenic treatment in patients with breast cancer. 

#### 2.3.5. Fibroblast Activation Protein (FAP)

**Figure 3:**
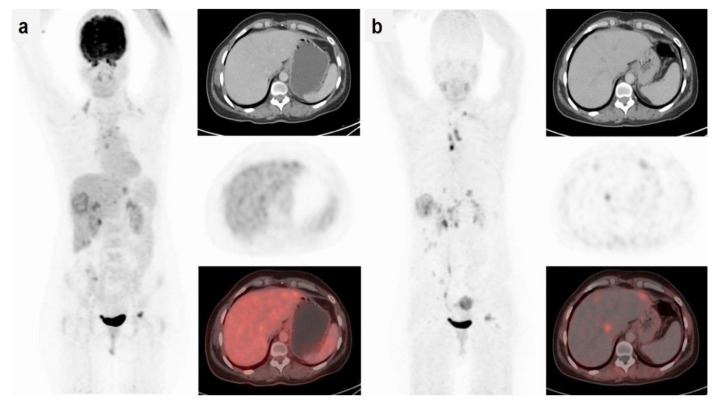
A 52-year-old patient with breast cancer. (**a**) [^18^F]FDG-PET/CT showed low or no uptake in the hepatic lesions (SUV_max_ = 3.9); (**b**) [^68^Ga]Ga-FAPI-04-PET/CT showed high uptake (liver metastases SUV_max_ = 9.1) in all lesions. (Reprinted with permission from Ref. [29], 2021, Springer Nature.)

FAP is overexpressed in cancer-associated fibroblasts in the tumor stroma of several types of cancers, including breast, colon, and pancreatic carcinomas. FAP plays a role in tumor invasion and metastasis [72]. The FAP inhibitor (FAPi) is currently being tested as a cancer therapeutic in clinical trials. Radiopharmaceuticals, such as [^68^Ga]Ga-FAPI-2 and [^68^Ga]Ga-FAPI-04, based on FAPi were found to be highly promising as molecular imaging probes [73]. [^68^Ga]Ga-FAPI-04-PET/CT and [^18^F]FDG-PET/CT were compared in 20 female breast cancer patients with primary and recurrent breast cancer in a prospective study [29]. PET/CT imaging with [^18^F]FDG and [^68^Ga]Ga-FAPI-04 were performed in the same week. In detecting primary breast lesions, [^68^Ga]Ga-FAPI-04 had a higher sensitivity than [^18^F]FDG (100% vs. 78.2%). PET/CT imaging with [^68^Ga]Ga-FAPI-04 also showed a significantly higher T/B ratio in breast lesions and in hepatic, bone, brain, and lung metastases (*p* < 0.05) (Figure 3). Thus, [^68^Ga]Ga-FAPI-04-PET may offer an advantage over [^18^F]FDG in delineating tumors to improve diagnosis and help guide FAPi therapy. Kömek et al. suggested that [^68^Ga]Ga-FAPI-04-PET has an advantage in detecting both primary and metastatic tumors because of its high sensitivity, high SUV_max_, and high T/B ratio. Limitations to this trial included the presence of latent bias due to the lack of histological verification on biopsied tissues. Kratochwil et al. found that these tracers could be useful beyond breast cancer [74]. FAP-targeted PET radiotracers are now considered as potential alternatives to [^18^F]FDG-PET [75,76]. 

### 2.4. Targeting Two Receptors Concurrently

The radiotracers discussed above only target one receptor. These strategies have some limitations. Some of these receptors may not be highly expressed in the tumor or its microenvironment relative to normal tissues. Some of the radiotracers might have relatively low binding affinities for their targets, or their pharmacokinetic properties in vivo may be suboptimal to achieve superior T/B ratios [77]. To overcome these limitations, several dual-receptor targeted radiotracers were developed in recent years. 

#### GRPR and Integrin αvβ3

To target both GRPR and integrin αvβ3, a heterodimeric peptide Glu-c(RGDyK)-bombesin (BBN-RGD) was synthesized and then radiolabeled with gallium-68 [78]. PET/CT imaging with [^68^Ga]Ga-BBN-RGD or [^68^Ga]Ga-BBN was conducted in 22 female patients with suspected breast cancer [30]. Imaging was performed at 30–45 min after injection. Eleven patients also underwent [^68^Ga]Ga-BBN-PET/CT within 2 weeks. [^68^Ga]Ga-BBN-RGD was taken up in both primary and metastatic lesions. For primary breast cancer, sensitivity was 95.8% and specificity was 60.0% for [^68^Ga]Ga-BBN-RGD. For lymph node metastases, sensitivity was 75.0% and specificity was 91.5% for [^68^Ga]Ga-BBN-RGD. [^68^Ga]Ga-BBN-RGD (SUV_max_ = 3.84 ± 2.18) showed better primary tumor detection with a higher SUV_max_, and higher sensitivity for both primary breast cancer and lymph node metastases than [^68^Ga]Ga-BBN (SUV_max_ =2.31 ± 0.72) (*p* < 0.05). In this study, the dual-receptor targeted [^68^Ga]Ga-BBN-RGD performed better than the mono-targeted [^68^Ga]Ga-BBN in diagnosing both primary and metastatic breast cancers. A comparison with [^68^Ga]Ga RGD-PET would be needed in future studies to truly assess the value of the dual-targeted probe. 

**Figure 4:**
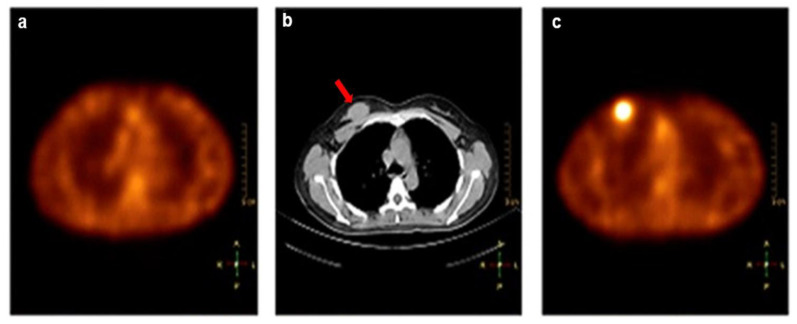
SPECT/CT images of a breast tumor that is positive for GRPR but negative for integrin αvβ3 expression. (**a**) [^99m^Tc]Tc-3P4-RGD2 SPECT had no tracer uptake in the lesion. (**b**) CT scan showed a mass in the right breast (arrow). (**c**) [^99m^Tc]Tc-RGD-BBN SPECT demonstrated high uptake in the lesion. (Reprinted from Ref. [31]).

[^99m^Tc]Tc-RGD-BBN, which targets both integrin αvβ3 and GRPR, was developed to improve tumor detection over mono-targeted imaging agents. This study explored the safety, pharmacokinetics, and biodistribution of [^99m^Tc]Tc-RGD-BBN in six healthy volunteers. Additionally, the diagnostic performance of [^99m^Tc]Tc-RGD-BBN was compared with that of [^99m^Tc]Tc-3P4-RGD2 in 6 female patients with metastatic breast cancer [31]. [^99m^Tc]Tc-RGD-BBN demonstrated clear uptake in 6 palpable lesions, and [^99m^Tc]Tc-3P4-RGD2 demonstrated clear uptake in 5 out of 6 lesions. By IHC analysis, expression of both αvβ3 and GRPR were found in 4 out of 6 cases. One case was only positive for GRPR, and another was only positive for αvβ3. [^99m^Tc]Tc-RGD-BBN would be useful in detecting malignant tumors that are negative for integrin αvβ3 but positive for GRPR expression, as these phenotypes would not be detected by [^99m^Tc]Tc-3P4-RGD2 (Figure 4). Due to the promising imaging results and lower effective radiation dose, [^99m^Tc]Tc-3P4-RGD2 may have the possibility of extending imaging applications to breast cancer screening.

## 3. Discussion and Perspectives

Noninvasive molecular imaging is critical for the development of novel approaches in precision medicine because it allows a comprehensive understanding of receptor status in individual tumor lesions within the same patient. This approach has the potential to predict and monitor responses to targeted therapies. The development of new probes that can image smaller lesions, especially in sites of metastases, is critical. In this review, we highlighted novel probes evaluated in clinical research that have potential for future clinical use in patient selection and/or in monitoring responses to targeted treatment in breast cancer (Table 2). 

Since hormone receptors play a key role in breast cancer, mapping hormone receptors is very important in clinical diagnosis and therapy. [^18^F]FES is an FDA-approved and established tracer for ER mapping in vivo. The adaptation of [^18^F]FES has been slow because of the need to educate ordering providers and to identify the specific clinical applications where it would be superior to the gold standard [^18^F]FDG-PET. We believe that [^18^F]4FMFES is a more promising tracer in breast cancer than [^18^F]FES, due to its lower overall background and higher sensitivity. 

Additionally, affibody molecules are well-studied as a probe in mapping HER2. The latest [^68^Ga]Ga-ABY-025 has solved the problem of high background activity in the liver that limited [^68^Ga]Ga-ABY-002 [18]. [^68^Ga]Ga-ABY-025 mitigated the limitations of antibody tracers, such as [^111^In]In-CHX-A″-DTPA-trastuzumab and [^64^Cu]Cu-DOTA-trastuzumab. Waiting several days after injection to achieve optimum T/B ratios may not be so convenient. However, high tracer uptake in the kidneys is still prominent. Tolmachev et al. thought that this high renal reabsorption is caused by the high affinity of scavenger receptors in the kidney to the affibody and is not dependent on target specificity [79]. Thus, the invention of affibody molecules with low renal reabsorption is key to improving imaging contrast of this class.

In addition, the successful application of CXCR4-directed theranostics in hematologic malignancies makes CXCR4 a promising target in other types of cancers [80,81]. The value of [^68^Ga]Pentixafor has previously been investigated in patients with esophageal and lung cancers [82,83]. Due to the heterogeneous accumulation of the CXCR4-targeted tracer and the small patient population, the [^68^Ga]Pentixafor study failed to clearly demonstrate its usefulness for cancer prediction, prognosis, or tumor grade of breast cancer. The complex biology of CXCR4 in breast cancer warrants further studies. In addition, the significant correlation between CXCR4 and HER2 expression opens the possibility of using [^68^Ga]Pentixafor to monitor response to HER2-targeted therapy.

Moreover, FAP is a very promising target for breast cancer. Elboga et al. showed the theranostic potential of [^68^Ga]Ga-FAPI in a retrospective study that included 48 patients with breast cancer [84]. Because of its high tumor-to-liver ratios, [^68^Ga]Ga-FAPI-PET/CT has the potential to delineate liver metastases of breast cancer and may provide an advantage over [^18^F]FDG-PET in metastatic disease. Actually, the clinical trials of FAP-targeting radiotherapy are ongoing in patients with different types of cancer, including nasopharyngeal carcinoma and pancreatic adenocarcinoma [85,86]. In addition to FAPI-02 or FAPI-04, another FAPi agent, DO-TA.SA.FAPi, has been developed and labeled with gallium-68 and lutetium-177 for evaluation in an end-stage breast cancer patient [87]. An intense radiotracer accumulation was noted in all the lesions by [^68^Ga]Ga-DOTA.SA.FAPi-PET/CT scans, and no treatment-related adverse events were observed. There is no doubt that the feasibility of [^68^Ga]Ga-FAPI will promote more theranostic approaches to FAP-targeted radiotherapy for breast cancer in the future.

Currently, several markers, including HER2, GRPR, and somatostatin receptors (SSTR), are being investigated as possible targets for targeted radionuclide therapy in the treatment of breast cancer [88,89]. The successful application of imaging probes such as [^68^Ga]Pentixafor and [^68^Ga]Ga-FAPI might lead to the development of variants for radionuclide therapy in the coming years. 

Almost all the clinical studies we reviewed have the limitation of small sample size. For example, only two patients were included in [^68^Ga]Ga-NOTA-MAL-Cys-MZ_HER2:342_ study, as this study was a first-in-human investigation. Most of the subjects in these studies are patients with breast cancer in different stages of disease. In the [^18^F]4FMFES study, only ER-positive breast cancer patients were involved. In some trials, only subjects with suspected breast cancer were enrolled, such as in the [^68^Ga]Ga-BBN-RGD trial. This factor probably contributed to the high rate of false-positive results. To get accurate evaluation about the sensitivity and specificity of each tracer, a larger sample size that includes a cohort of women with inconclusive results on mammographic evaluation is needed in future clinical studies. Thus, more factors need to be considered and more rigorous clinical validations are required before a novel tracer can be adopted in clinical application. 

For the quantification of radiotracers in vivo, SUV values are still the main calculated endpoints in clinical practice, since kinetic modeling requires a good deal of time and expertise. In some conditions, such as subcentimeter metastatic lesions in lymph nodes, liver, and bone, kinetic modeling would be more informative than SUV in mapping lesions through a more accurate quantification of tracer uptake. Currently, kinetic modeling is not used in any of the clinical Picture Archiving and Communication System (PACS) workflows because of its complexity. There is an increase in the use of machine learning tools in PACS, but incorporation of kinetic modeling has not yet been implemented [90]. This is an exciting area of development, and we foresee many new applications of PACS-based analytic tools, such as machine learning applications and kinetic modeling, in the next 10 years.

With the development of new targeted treatments, new imaging agents that can predict or monitor the response to treatment have the potential to impact precision medicine. Above all the tracers we reviewed, [^68^Ga]Ga-FAPI-04-PET/CT is now undergoing clinical trials in patients across many different types of cancer. We believe that this tracer would have a great possibility to be the next imaging agent to use in the clinic. However, imaging of hormone receptors, integrin receptors, angiogenesis biomarkers, gastrin receptors, and fibroblast-associated protein provide promising strategies for progressing precision medicine. Defining the roles of these imaging biomarkers in the context of targeted therapy will aid in the adoption of these tracers in clinical practice. We hope that more imaging agents with higher precision, sensitivity, and specificity for breast cancer patients will be developed in the near future. 

## 4. Conclusions

Many advanced, safe, efficient, and noninvasive imaging agents have been successfully applied in clinical investigations for breast cancer patients as first-in-human studies. These noninvasive imaging approaches will contribute to advance precision medicine for breast cancer patients, not only to improve tumor detection in primary or metastatic lesions, but also to help guide targeted treatment in this heterogeneous disease. These studies are important first steps toward larger clinical trials to identify the best PET or SPECT imaging agents, to shift paradigms in clinical practice to precision medicine. 

## Figures and Tables

**Table 1:** Properties of targeted nuclear imaging agents for breast cancer.

Target	Imaging Agent	Type of Probe	Imaging Modality	Method of Quantification	References
HER 2	[^111^In]In-CHX-A”-DTPA-trastuzumab	Antibody	SPECT	T/B	[15]
	[^68^Ga]Ga-NOTA-MAL-Cys-MZHER_2:342_	Affibody	PET	SUV_max_	[16]
	[^64^Cu]Cu-DOTA-trastuzumab	Antibody	PET	SUV_max_	[17]
	[^68^Ga]Ga-ABY-025	Affibody	PET	Kinetic modeling and SUV	[18]
	[^99m^Tc]Tc-(HE)3-G3	Protein	SPECT	T/B	[19]
ER	[^99m^Tc]Tc-tamoxifen	Small Molecule	SPECT	T/B	[20]
	[^18^F]4FMFES	Small Molecule	PET	SUV_max_	[21]
PR	[^18^F]FFNP	Small Molecule	PET	SUV_max_	[22]
AR	[^18^F]FDHT	Small Molecule	PET	SUV_max_	[23]
Integrin αvβ3	[^99m^Tc]Tc-3PRGD_2_	Peptide	SPECT	T/B	[24]
GRPR	[^68^Ga]Ga-RM2	Peptide	PET	SUV_max_	[25]
	[^68^Ga]Ga-NOTA-RM26	Peptide	PET	SUV_max_	[26]
CXCR4	[^68^Ga]Pentixafor	Peptide	PET	SUV_max_ and T/B	[27]
PSMA	[^68^Ga]Ga-PSMA-HBED-CC	Peptide	PET	SUV_mean_	[28]
FAP	[^68^Ga]Ga-FAPI-04	Small molecule	PET	SUV_max_	[29]
GRPR and Integrin αvβ3	[^68^Ga]Ga-BBN-RGD	Bispecific peptide	PET	SUV_mean_	[30]
	[^99m^Tc]Tc-RGD-BBN	Bispecific peptide	SPECT	T/B	[31]

Legend: SPECT = single photon emission computed tomography; PET = positron emission tomography; SUV = standardized uptake value; T/B = tumor-to-background ratio.

**Table 2:** Summary of clinical evaluation of nuclear imaging agents in breast cancer.

Target	Agent	Phase Study	Study Population	Number of Patients	Key Results
HER 2	[^111^In]In-CHX-A”-DTPA-trastuzumab	Phase 0	Metastatic Breast Cancer	11	− Administration to humans was safe − Sensitive for imaging HER2 expression
	[^68^Ga]Ga-NOTA-MAL-Cys-MZHER_2:342_	N/A	Breast cancer	2	− Monitored HER2 levels in breast cancer − Low background in liver
	[^64^Cu]Cu-DOTA-trastuzumab	N/A	HER2-positive metastatic breast cancer	8	− Visualized HER2-positive metastatic breast cancer with high sensitivity
	[^68^Ga]Ga-ABY-025	N/A	Metastatic breast cancer	16	− Tracer kinetic modeling can be used to evaluate metastatic HER2 expression accurately
	[^99m^Tc]Tc-(HE)3-G3	Phase 1	Primary breast cancer	28	− Administration to humans was safe − Delineated HER2-positive and HER2-negative breast cancer.
ER	[^99m^Tc]Tc-tamoxifen	N/A	ER-positive breast cancer	1	− Identified the active tumor and visualize ER-positive sites
	[^18^F]4FMFES	Phase 2	ER-positive breast cancer	31	− Lower background activity than [^18^F]FES − Better tumor contrast than [^18^F]FES
PR	[^18^F]FFNP	Phase 2	ER-positive Breast Cancer	43	− SUV_max_ change of [^18^F]-FFNP after estradiol challenge is highly predictive of response to endocrine therapy in ER-positive breast cancer patients
AR	[^18^F]FDHT	Phase 2	ER positive metastatic breast cancer	10	− Relatively low interobserver visual agreement, but good quantitative agreement compared to [^18^F]FES
Integrin αvβ3	[^99m^Tc]Tc-3PRGD2	N/A	Breast cancer	42	− Less sensitive in detecting small lymph node metastatic lesions than [^18^F]FDG
GRPR	[^68^Ga]Ga-RM2	N/A	Primary breast cancer	15	− SUV_max_ of [^68^Ga]Ga-RM2-PET correlated with ER expression in primary tumors of untreated patients
	[^68^Ga]Ga-NOTA-RM26	Early Phase 1	Breast cancer	35	− SUV_max_ of [^68^Ga]Ga-NOTA-RM26-PET in breast cancer correlated with ER expression and menstrual status of the patient.
CXCR4	[^68^Ga]Pentixafor	N/A	Primary and recurrent breast cancer Breast metastases of unknown primary	18	− Feasible to detect primary and recurrent breast cancer − Tumor detectability was significantly lower than that of [^18^F]FDG-PET
PSMA	[^68^Ga]Ga-PSMA-HBED-CC	N/A	Breast cancer	19	− Higher uptake in distant metastases than in primary tumor − Confirmed the reported variation of PSMA expression
FAP	[^68^Ga]Ga-FAPI-04	N/A	Primary and Recurrent breast cancer	20	− Superior to [^18^F]FDG in detecting primary tumor and metastatic lesions in lymph node, liver, bone, and brain
GRPR and Integrin αvβ3	[^68^Ga]Ga-BBN-RGD	Phase 1	Breast cancer	22	− SUV_mean_ of [^68^Ga]Ga-BBN-RGD-PET correlated well with both GRPR expression and integrin αvβ3 expression in primary and metastatic lesions
	[^99m^Tc]Tc-RGD-BBN	N/A	Metastatic breast cancer	22	− Administration to humans was safe − More sensitive in the detection of breast cancer with only GRPR positive expression than [^99m^Tc]Tc-3P4-RGD2
